# Progress in the application of virtual reality technology in the rehabilitation of patients with pusher syndrome after stroke

**DOI:** 10.3389/fneur.2026.1769598

**Published:** 2026-02-17

**Authors:** Junsheng Hao, Fuxian Lv, Haochong Song, Haoyang Duan

**Affiliations:** 1 Department of Rehabilitation Medicine, The People's Hospital of Dongchang District, Tonghua, China; 2 Department of Rehabilitation Medicine, First Hospital of Jilin University, Changchun, China; 3 College of Special Education, Beijing Union University, Beijing, China

**Keywords:** postural control, pusher syndrome, rehabilitation, stroke, virtual reality technology, visual feedback

## Abstract

Pusher syndrome (PS) after stroke is a postural control disorder characterized by an abnormal perception of verticality, where patients persistently lean toward the hemiplegic side and resist postural correction, severely affecting balance function and rehabilitation progress. With its immersive, interactive, and programmable features, virtual reality (VR) technology offers new approaches for the rehabilitation of PS, including multisensory integration, real-time feedback, and personalized training. This article reviews the clinical features, pathological mechanisms, and rehabilitation challenges of PS, and systematically elaborates on the advantages of VR technology in correcting perceptual deviations, improving postural control, and enhancing treatment motivation and compliance through functions such as visual-postural recalibration, body schema reconstruction, and balance task training. Additionally, it analyzes the challenges faced by VR technology in clinical application, including hardware discomfort, insufficient standardization of protocols, uncertain cost-effectiveness, and limited evidence of long-term efficacy. Future research should focus on device optimization, protocol standardization, high-level evidence-based validation, and technology integration to promote the precise, personalized, and widespread application of VR in the rehabilitation of PS.

## 1 Clinical features of pusher syndrome

Pusher syndrome (PS) is a clinical syndrome primarily characterized by severe postural control impairment, commonly observed in patients following stroke or other central nervous system injuries (1–4). First systematically described by Davies in 1985, its core clinical feature is the patient’s inability to maintain a normal upright or seated posture, with the body persistently leaning toward the hemiplegic side (5). Notably, even with external assistance, patients often exhibit significant resistance to postural correction. This active resistance phenomenon complicates and adds challenge to rehabilitation interventions (6, 7).

Regarding the incidence of PS, existing research shows some variation, which may be related to differences in research methodologies, sample characteristics, and assessment criteria. In his initial report, Davies indicated an incidence of approximately 25% among stroke patients (5), while data from the Copenhagen Stroke Institute in 1996 showed an incidence ranging from 5 to 10% (8). Despite these variations, most studies suggest the syndrome’s incidence in stroke patients is around 10 to 25%, indicating it is relatively common among post-stroke dysfunctions, with a higher occurrence particularly in patients with recurrent stroke or severe brain injury (9–12). Differences in incidence may also be influenced by factors such as age, gender, timing of rehabilitation intervention, and its effectiveness. Inconsistencies in diagnostic criteria across different studies may also be a reason for the data fluctuations (13).

The clinical manifestations of PS are characteristic (14). In the supine position, patients display a typical posture of tilting toward the affected side due to trunk asymmetry, accompanied by compensatory head rotation toward the unaffected side (15). In the seated position, significant trunk tilt toward the affected side is evident, with weight primarily borne by the buttocks on that side and the head also rotated toward the unaffected side, further affecting sitting balance (16). Patients experience particular difficulty in shifting their weight toward the unaffected side during dynamic activities like weight shifting or trunk rotation (17). While standing, the center of gravity shifts toward the affected side, and some patients rely on external support to maintain balance (18). During walking, patients exhibit impaired weight shifting and unsteady gait, accompanied by a compensatory posture of head rotation toward the unaffected side and trunk tilt toward the affected side (19).

Regarding prognosis, research indicates that PS may significantly prolong the rehabilitation process, especially in the acute phase, but it typically does not affect the ultimate functional recovery (20). Prognostic influencing factors include the etiology (e.g., recovery is often slower in cases caused by traumatic brain injury or tumors), the site of the lesion (recovery is often slower with right-brain lesions compared to left-brain lesions), and the presence of comorbidities such as proprioceptive or visuospatial neglect (21). Danells et al. (11) research further suggests a significant correlation between the duration of PS and unilateral spatial neglect. Long-term prognostic studies have yielded inconsistent results: Karnath et al. (22) reported that approximately 90% of patients recover substantially within 6 months post-stroke, with favorable long-term functional outcomes. However, other follow-up studies have found that about 10–15% of patients still have residual symptoms 2 years post-stroke, which may persistently affect activities of daily living (20). Systematic rehabilitation therapy can help improve patients’ postural control, balance function, and activities of daily living, enhance quality of life, and reduce the risk of complications. Therefore, establishing an early diagnosis system and implementing personalized rehabilitation programs are of significant importance for optimizing the prognosis of patients with PS (13).

## 2 Pathological mechanisms of PS

The pathogenesis of PS is not yet fully elucidated, and it is widely recognized in academia as a central postural perception disorder resulting from the interaction of multiple factors (23). Current research suggests its occurrence may be closely related to functional abnormalities in the following aspects: Firstly, dysfunction of the vestibular system post-stroke may lead to abnormal processing of gravitational perception signals, causing patients to misinterpret the vertical direction (24). Secondly, abnormal proprioceptive input results in the misinterpretation of pressure and positional signals from the unaffected side of the body, triggering the illusion of being “pushed from the unaffected side” (25). Furthermore, motor control impairment manifests as the patient’s inability to execute appropriate postural adjustment strategies based on correct spatial information. Damage to higher cortical integration functions, particularly involving the parietal-insular-thalamic network, is considered a key mechanism affecting sensory information integration and the formation of correct body spatial representation (26).

The maintenance of human balance and postural control primarily relies on two key sensory pathway systems: the subjective postural vertical (SPV) pathway and the subjective visual vertical (SVV) pathway (27). The SPV pathway integrates information from the vestibular system, proprioceptors, and pressure receptors to form an internal perception of the body’s spatial position. The SVV pathway, through processing visual information, establishes an accurate judgment of the vertical direction of the external environment. Under physiological conditions, these two pathways are integrated and coordinated by the central nervous system to form a unified vertical reference frame, enabling individuals to accurately perceive and maintain an upright posture and ensuring the precision of balance control and motor coordination. This multisensory integration mechanism is not only the neural basis for postural control but also plays a vital role in maintaining activities of daily living and motor function.

Following a stroke, the neural pathways related to the subjective postural vertical and subjective visual vertical may be damaged, which is an important pathological basis for PS (28). Essentially, this syndrome is a spatial cognitive disorder originating from the brain’s misinterpretation of the vertical spatial reference frame. This misinterpretation is closely related to the integration dysfunction between visual information and postural sense input (29). Specifically, damage to the SPV pathway can lead to vestibulo-proprioceptive integration dysfunction, preventing patients from accurately perceiving the positional relationship between their body’s central axis and the direction of gravity. This spatial perception abnormality manifests as a tendency for patients to lean toward the hemiplegic side and exhibit resistance reactions during passive postural correction. These clinical features are significantly associated with distortions in the spatial representation of the affected side.

Numerous studies have demonstrated a clear correlation between deviations in the subjective postural vertical and PS. The case study by Romick-Sheldon et al. (30) was the first to systematically reveal that the direction of the subjective postural vertical deviation in PS patients is consistent with their postural tilt direction, both pointing toward the side contralateral to the lesion, The individual’s SCP score decreased from 3.75 out of 6 to 0.75 out of 6 during the course of the intervention (30). Pérennou et al. further confirmed this finding through a longitudinal study and were the first to report that the recovery of subjective postural vertical orientation ability is temporally synchronized with the clinical improvement of PS symptoms. Of six patients with brainstem stroke and ipsilesional lateropulsion only one had an abnormal ipsilesional postural vertical tilt (6°); six had an ipsilesional visual vertical tilt (13 ± 0.4°); two had ipsilesional haptic vertical tilts of 6° (31). Mansfield et al. (32) research, using quantitative assessment methods, confirmed a dose-effect relationship between the degree of recovery in subjective postural vertical perception and the alleviation of PS symptoms. The study showed that as patients’ vertical perception ability gradually recovered, their core symptoms such as postural tilt and resistance to correction also improved accordingly. Subjective visual vertical was significantly more biased toward the contralesional side for participants with history of pushing (−3.6. ±. 4.1°) than those without (−0.1. ±. 1.4°) (32). This not only establishes the key role of the subjective postural vertical in the pathological mechanism but also provides a theoretical basis for clinical rehabilitation.

However, there remains significant controversy regarding the relationship between the subjective visual vertical and PS. Through experiments strictly controlling visual input, Karnath et al. (33) found that when visual information was excluded, PS patients exhibited significant spatial perception deviations, with their subjective vertical perception averaging an offset of about 18° toward the lesioned side, suggesting a possible specific impairment in the postural vertical perception system. The study by Paci et al. (34) further supports this view, as they found that providing visual feedback could effectively correct patients’ postural deviations, indicating that their visual vertical perception system is relatively intact, while the postural vertical perception pathway may have selective functional impairment.

These findings not only challenge the traditional single-pathway impairment theory but also indicate that the pathological mechanisms of PS may involve complex interactions among multiple sensory systems. Therefore, future research needs to employ multimodal methods combined with advanced neuroimaging techniques to further elucidate the roles and interrelationships of various sensory pathways in the mechanisms of PS.

## 3 Theoretical basis for the application of virtual reality technology

The rehabilitation of patients with PS faces multiple challenges, primarily including correcting vertical perception deviations, reconstructing normal body schema and spatial reference frames, optimizing postural control and balance response capabilities, and enhancing safe transfer and mobility during functional activities (35). These goals involve the multi-dimensional integration of sensory, cognitive, and motor systems and require patients to perform continuous postural adjustments in dynamic environments. However, traditional rehabilitation methods often struggle to balance comprehensive needs such as multi-sensory stimulation, real-time feedback, personalized adjustments, and safety, leading to limited training effects and low patient compliance (13).

Virtual Reality (VR) technology provides theoretical support and a practical pathway for these rehabilitation needs.

### 3.1 Correcting vertical perception deviation

Due to central nervous system damage, patients often experience subjective postural vertical deviation, resulting in a persistent tilt of the body toward the affected side. VR technology can guide patients to gradually perceive and adjust their internal vertical reference frame by setting virtual visual vertical reference lines, dynamic tilting scenes, and real-time center-of-gravity feedback. The system can immediately display the posture deviation angle and, through visual cues or task-oriented gamification mechanisms, motivate patients to actively correct their posture, thereby progressively rebuilding correct vertical perception ability (36).

### 3.2 Reconstructing body schema and spatial reference frames

The reconstruction of body schema and spatial reference frames relies on the synergistic integration of multimodal sensory inputs. VR systems can integrate visual, vestibular, proprioceptive, and tactile information to simulate real spatial relationships and body position changes within virtual environments (37). For example, by adjusting optical flow speed, ground slope, and obstacle layout in virtual walking tasks, neural mechanisms related to the patient’s spatial orientation and body schema can be activated. Immersive experiences can also enhance the sense of embodiment, encouraging patients to identify the virtual body as part of themselves, thereby facilitating the remodeling of the body schema.

### 3.3 Improving postural control and balance response capabilities

Enhancing postural control and balance function depends on high-intensity, repetitive, and task-oriented training. VR can provide diverse balance challenges, such as virtual surfing, stepping-stone river crossing, and obstacle avoidance walking. The system can dynamically adjust task difficulty based on patient performance, achieving personalized training. Simultaneously, by real-time monitoring of center-of-gravity trajectory, gait parameters, and joint movement, VR can provide immediate visual or auditory feedback, assisting patients in optimizing postural control strategies and enhancing anticipatory and reactive balance capabilities (38).

### 3.4 Promoting safe transfer and mobility

To improve patients’ transfer and mobility abilities in real environments, VR can simulate daily life scenarios such as kitchens, supermarkets, and bus stops, and set up various situations like changing support surfaces, moving crowds, or sudden obstacles to train their ability to cope with complex environments (39). The training process can be conducted with the support of standing frames, safety harnesses, or robotic assistance, enhancing patient confidence and functional adaptability while ensuring safety.

From the perspective of neurorehabilitation mechanisms, VR holds significant theoretical value in the following aspects.

#### 3.4.1 Perceptual-cognitive level

Through first-person virtual avatars and synchronized movement feedback, VR can enhance the sense of body ownership and agency, directly acting on the impaired body schema and assisting patients in recalibrating distorted spatial references and vertical perception (40).

#### 3.4.2 Neuroplasticity level

VR can create high-intensity, highly repetitive, task-rich, and engaging training environments. Such “dose-intensive” training paradigms have been proven to promote functional reorganization and synaptic strengthening in the sensorimotor cortex and related neural networks, providing a neurobiological basis for motor function recovery (41).

#### 3.4.3 Behavioral transfer level

VR environments offer good customizability and ecological validity, supporting targeted training in simulated daily scenarios. By repeatedly practicing correct postural adjustments, weight shifting, and balance strategies in the virtual environment, patients can internalize the learned motor patterns and promote their transfer and generalization to real-life situations (42).

**Figure 1:**
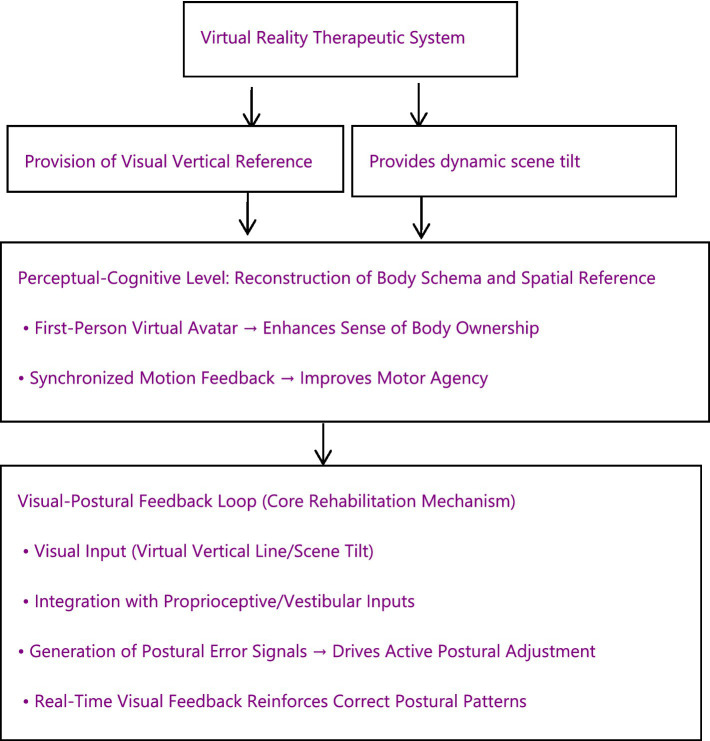
A conceptual framework figure illustrating the visual-postural feedback loop.

Leveraging its immersion, interactivity, and programmability, VR technology constructs an integrated rehabilitation platform for patients with PS, encompassing perceptual remodeling, motor relearning, and functional transfer. This technology can not only address the core pathological mechanisms of PS but also effectively enhance patient engagement and rehabilitation motivation. Consequently, it provides an ideal technological pathway for achieving personalized, efficient, and safe rehabilitation interventions (Figure 1).

## 4 Methods

This study followed the Preferred Reporting Items for Systematic Reviews and Meta-Analyses (PRISMA) guidelines and was conducted by two independent reviewers, who systematically reviewed literature published from January 2000 to December 2025. The included studies were all written in English, focused on human subjects, and aimed to analyze the effectiveness of virtual reality technology in the rehabilitation of patients with Pusher Syndrome (PS) following stroke. The literature search was conducted in the specialized databases MEDLINE, Scopus, PEDro, and Web of Science, using the following keyword combination: (“Pusher Syndrome” OR “Pushing Behavior” OR “Contraversive Pushing”) AND (“Treatment” OR “Virtual Reality” OR “Rehabilitation Therapy”).

1. The inclusion criteria based on the PICO framework are as follows: ① Study types: Randomized controlled trials, case series studies, case reports, and pilot studies; ② Study participants: Adult patients diagnosed with Pusher Syndrome after stroke, regardless of gender or specific age; ③ Interventions and comparisons: Virtual reality technology as the core intervention, either alone or combined with other rehabilitation measures. Other technologies may serve as comparators, or no comparator may be used (applicable to case series studies); ④ Outcome measures: Study results must be assessed using specific and validated evaluation tools or scales.
2. Exclusion criteria: Studies that only used assessment scales without implementing virtual reality rehabilitation interventions.

In the randomized clinical trials, the methodological quality of the selected studies was assessed using the CASPe33 guidelines. Although case series studies have a relatively limited level of evidence, they were still included in the analysis due to the scarcity of relevant research in this thematic area. The aim was to integrate supplementary information from pre-experimental studies, whose protocols could provide methodological references for future clinical trials.

The level of evidence and strength of recommendations were evaluated based on the classification criteria established by the Oxford Centre for Evidence-Based Medicine (CEBM). This system is one of the most widely used evidence grading frameworks in the field of health. Its strength lies in its ability to offer a highly specialized evidence classification structure tailored to the characteristics of different clinical scenarios.

## 5 Application of VR technology

Visual feedback training is a rehabilitation therapy technique based on the principles of neuroplasticity. It promotes neural pathway reorganization and compensatory recovery of motor function by providing patients with real-time visual information, thereby activating the brain’s motor cortex and mirror neuron systems. Its mechanism encompasses multiple neurophysiological processes, including visual information perception and processing, motor intention formation, and cognitive regulation of motor execution (43). In the rehabilitation of stroke patients with hemiplegia, visual feedback training has been confirmed to significantly improve motor control and enhance activities of daily living.

In interventions for PS, visual feedback training primarily leverages the crucial role of the visual system in motor control and postural adjustment. It aims to improve symptoms by enhancing patients’ proprioceptive integration and spatial orientation abilities. Specifically, therapists can provide external references for body position and movement trajectories to patients using mirrors, real-time video, or computer-assisted visual cues. This visual information helps patients reconstruct correct body spatial representation and correct abnormal postural control patterns (44), thereby gradually restoring normal postural adjustment mechanisms. Research indicates that such visual feedback-based interventions can effectively improve trunk tilt behavior and postural control in patients with PS.

Traditional visual feedback training often uses mirrors as feedback tools, widely applied in clinical practice due to their simplicity and low cost. Hwang et al.’s study systematically evaluated the efficacy of mirror feedback training for patients with PS, showing that this method could significantly reduce patients’ SCP scores. This type of training is not only simple in process but also easy to standardize, facilitating clinical promotion (45).

With the development of computer technology, computer-based visual feedback systems are gradually replacing traditional mirror feedback. Compared to mirror feedback, computer-generated interactive visual feedback systems can provide real-time, quantifiable, and recordable postural information, enabling more precise training design. Yang et al. (18) noted that functional impairments in PS patients often involve multiple anatomical planes, whereas traditional mirror feedback cannot provide quantitative data. In contrast, computer systems can provide quantitative postural information across multiple dimensions such as the frontal, sagittal, and horizontal planes, demonstrating significant advantages (18).

Both traditional mirror visual feedback and computer-based visual feedback systems play positive roles in improving patients’ postural control ability, thereby significantly enhancing their quality of life. Although computer systems have evident advantages in therapeutic effect and precision, their higher equipment costs currently limit widespread adoption in clinical and home environments. In contrast, mirror visual feedback technology, due to its simplicity and lower cost, is more suitable for home-based rehabilitation training for patients with PS.

Clinical studies have shown that virtual reality technology can provide patients with continuous visual feedback, effectively guide them to maintain correct postures, and promote central neural remodeling. Research by Kim et al. confirmed that virtual reality technology, through real-time visual feedback mechanisms, not only assists patients in correcting abnormal postures but also accelerates the process of neuroplasticity, thereby enhancing rehabilitation outcomes (19). Furthermore, the approach combining visual guidance with core stability training has also been proven to effectively improve patients’ tilted postures and enhance vertical perception.

Building on previous research, some scholars have explored the combined application of virtual reality and robotic technology. This technology enables high-repetition training and utilizes harness assistance to facilitate early upright posture training, demonstrating its efficacy as superior to traditional physical therapy combined with visual feedback (46, 47). However, the lack of standardized definitions for the therapist’s role makes it difficult to distinguish whether the therapeutic effects stem from the technology itself or the combined effects of human interaction, resulting in ambiguous attribution of outcomes and challenges in replicating the protocols.

Regarding direct clinical evidence for Pusher syndrome, existing research is extremely limited. A single-case study by Nestmann et al. (48) found that presenting patients with a 20° tilted virtual reality scene significantly reduced their tilting behavior, and the effects persisted post-intervention. This outcome supports the “visual-postural recalibration” theory and provides preliminary direct clinical evidence for virtual reality tilt therapy (48). However, as a single-case report, the evidence level is low, and further validation through randomized controlled trials with larger sample sizes is required.

In terms of indirect supporting evidence, Wöhrstein et al. (49) developed a “tilt reality device” that can display tilted views of the surrounding environment in real time. This device demonstrated good tolerability in healthy populations, with an average usage time of approximately 40 min, providing a feasibility basis for future long-term application in patients with Pusher syndrome (49). However, it is important to note that this study only included healthy adults and is a preclinical usability study; its conclusions cannot be directly extrapolated to the clinical efficacy evaluation of post-stroke Pusher syndrome patients.

The current evidence system for virtual reality technology in treating Pusher syndrome exhibits distinct stratification. Clinical studies directly targeting Pusher syndrome patients are scarce, with generally low levels of evidence. In contrast, indirect evidence from healthy populations or other stroke patients with balance disorders is relatively abundant, providing methodological references and technical feasibility support for subsequent research. However, such evidence cannot substitute for direct clinical efficacy data. Future research should prioritize high-quality randomized controlled trials targeting Pusher syndrome patients to clarify the true clinical effects of virtual reality technology (Table 1).

**Table 1:** Table of included studies.

References	Author/year	Study design (Case/RCT)	Sample size (*N*)	Intervention protocol	Key findings
(45)	Hwang KK/2011	Case report	1	Visual feedback and body posture control training	The SCP score changed from 3.75 points to 0.8 point
(18)	Yang YR/2015	RCT	12	Computer-generated interactive visual feedback training	the program more effectively aided recovery from pusher syndrome
(19)	Kim MS/2016	RCT	10	Robot-assisted rehabilitation with virtual reality	improvement of balance and gait function
(46)	Krewer C/2013	RCT	25	Physiotherapy with visual feedback components	a significant effect on the BLS，but no significant effect on the SCP
(47)	Yun N/2018	RCT	36	Robot-assisted gait training (RAGT)	RAGT ameliorated lateropulsion and balance function more effectively
(48)	Nestmann S/2022	Case report	1	Tilted 3D visual input	the program reduce symptoms of severe contraversive lateropulsion
(49)	Wöhrstein S/2025	RCT	36	Tilted Reality Device (TRD)	TRD is especially for an older population

## 6 Advantages and challenges of VR technology application

### 6.1 Application advantages

#### 6.1.1 Controllable multi-sensory feedback environment for precise correction of perceptual deviations

VR technology allows for precise control over the presentation of visual flow, proprioceptive cues, and the gravitational reference frame. This enables therapists to personalize the setting of the “vertical direction” within the virtual environment according to the degree of the patient’s perceptual deviation. By gradually adjusting the discrepancy between the virtual visual vertical line and the actual gravitational direction, patients can be guided to reconstruct a correct spatial perception framework. Furthermore, VR can enhance or suppress input from specific sensory channels (e.g., by strengthening visual feedback to compensate for impaired proprioception), promoting multisensory integration and recalibration, and achieving precise interventions that are difficult to accomplish with traditional physical therapy.

#### 6.1.2 Safe, immersive training environment supporting high-difficulty balance challenges

Within a completely safe virtual environment, patients can perform balance training activities that carry a high risk of falls in reality, such as standing on a swaying platform or walking across a narrow virtual bridge. This “failure-permissive” environment significantly reduces patients’ fear and anxiety, encouraging them to actively explore their motor limits and attempt weight shifts and postural adjustments that are difficult to execute in reality, thereby more effectively overcoming functional limitations (50).

#### 6.1.3 Gamification and task-oriented design to enhance treatment motivation and compliance

The rehabilitation process for PS is often lengthy and monotonous. VR can transform repetitive balance and posture correction exercises into goal-oriented, immediate-feedback, reward-based gaming tasks (e.g., “catch flying balls to score points,” “move the center-of-gravity cursor out of the danger zone”). Such gamified designs can effectively stimulate patients’ intrinsic motivation, improve their training focus, duration, and enjoyment, thereby ensuring the necessary “therapeutic dosage” for rehabilitation and enhancing long-term compliance.

#### 6.1.4 Objective quantification and real-time monitoring supporting precise assessment and dynamic adjustment

VR systems can continuously and in real-time record kinematic parameters during patient training, such as body tilt angle, center-of-gravity sway trajectory, postural adjustment reaction time, and task completion accuracy. These objective data provide therapists with far more detailed information than traditional scale assessments, helping to quantify the degree of improvement in perceptual deviations. Training difficulty and focus can be dynamically adjusted based on this data, enabling a truly personalized rehabilitation pathway (51).

### 6.2 Challenges faced

#### 6.2.1 Hardware technology and sensory conflict-induced physical discomfort

Some patients may experience symptoms such as motion sickness, visual fatigue, or headaches when using head-mounted displays. For PS patients with already impaired vestibular function, the mismatch between virtual visual motion and real proprioception may exacerbate discomfort or even induce dizziness, thereby limiting device usage time and effectiveness. Additionally, the weight, comfort, and ease of donning/doffing the equipment can affect patient acceptance, especially among the elderly or those with upper limb dysfunction (49).

#### 6.2.2 Complexity of personalized protocol design and lack of standardized protocols

Although VR offers high adjustability, there is currently a lack of standardized protocols based on high-level evidence-based medicine to guide the design of training parameters (e.g., degree of visual distortion, type of feedback, progression of task difficulty) for patients with different severity levels, injury mechanisms, and rehabilitation stages (48). Therapists need to master both rehabilitation knowledge and VR technology, which presents a certain barrier to clinical adoption.

#### 6.2.3 Insufficient evidence-based proof for long-term efficacy and skill transfer

Although preliminary studies indicate positive effects from VR interventions, its long-term efficacy on functional recovery in patients with PS and its cost-effectiveness compared to conventional therapies still require validation through more large-sample, multicenter randomized controlled trials. Particularly crucial is the extent to which skills acquired in the virtual environment can be stably transferred to complex and variable real-world situations, which remains a core issue in current research and practice.

#### 6.2.4 Patient individual differences and scope of application limitations

Not all patients with PS are suitable for VR intervention. Patients with severe cognitive impairment, visual deficits, significant spasticity, or extreme anxiety may be unable to effectively participate or benefit. Therefore, rigorous patient screening and assessment are necessary to ensure the suitability and safety of the technology’s application.

## 7 Summary

The core pathological features of PS are abnormal perception of verticality and dysfunction in multisensory integration. Leveraging its immersive, interactive, and programmable characteristics, VR technology can create a highly controllable, multisensory-coordinated rehabilitation environment, enabling precise correction of patients’ perceptual deviations and systematic training of postural control capabilities. Existing studies confirm that VR holds significant advantages in providing real-time visual feedback, supporting high-intensity balance training in safe environments, and enhancing patient motivation and treatment compliance. However, the clinical adoption of this technology still faces multiple challenges, including poor hardware tolerance, insufficient standardization of treatment protocols, unclear cost-effectiveness, and limited evidence of long-term efficacy.

To facilitate the clinical translation and optimization of VR technology for the rehabilitation of Pusher syndrome, future studies should adopt more operational designs. It is recommended to conduct multicenter randomized controlled trials, with sample sizes determined through power analysis based on preliminary data (e.g., assuming a moderate effect size of 0.25, *α* = 0.05, *β* = 0.20), ensuring adequate sample sizes (e.g., at least 50 cases per group). Outcome measures should integrate both clinical and mechanistic perspectives: the Burke Lateropulsion Scale is recommended as the primary outcome to assess postural deviation, while secondary measures should include the Subjective Visual Vertical Scale to quantify perceptual recalibration, supplemented by neuroimaging indicators (e.g., fNIRS-based brain network connectivity) to explore neuroplasticity mechanisms. To evaluate the sustainability of therapeutic effects, follow-up assessments at 3 and 6 months post-intervention should be included to monitor potential functional decline. Building on this foundation, research can progressively advance toward hardware miniaturization, protocol standardization, and technological integration, aiming to develop a more effective, accessible, and personalized intelligent rehabilitation system.
